# Evaluation of Propolis and Diclofenac Sodium Eye Drops for Animals: Physicochemical Properties and In Vitro Biological Activity

**DOI:** 10.3390/gels12070651

**Published:** 2026-07-20

**Authors:** Dovilė Svetikienė, Vita Ambrulaitienė, Gintarė Jančiukė, Ieva Sarapinienė, Gintaras Zamokas, Aidas Grigonis, Oleksandr O. Nefodov, Kristina Ramanauskienė

**Affiliations:** 1 Department of Dr. L. Kriauceliunas Small Animal Clinic, Faculty of Veterinary Medicine, Lithuanian University of Health Sciences, Tilzes Str. 18, LT-47181 Kaunas, Lithuania; gintaras.zamokas@lsmu.lt (G.Z.); aidas.grigonis@lsmu.lt (A.G.); 2 Institute of Cardiology, Lithuanian University of Health Sciences, 50162 Kaunas, Lithuania; vita.ambrulaitiene@lsmu.lt (V.A.);; 3 Department of General and Clinical Pharmacy, Odessa I.I. Mechnikov National University, 2 Vsevoloda Zmiienka St., Odesa 65082, Ukraine; o.nefodov@onu.edu.ua; 4 Department of Clinical Pharmacy, Faculty of Pharmacy, Lithuanian University of Health Sciences, Sukileliai Avenue 13, LT-50162 Kaunas, Lithuania

**Keywords:** eye, natural products, propolis, SIRC cell, ophthalmic gels, cytotoxic, flow cytometry, in vitro

## Abstract

Eye diseases are common in veterinary clinical practice and are most often treated with topical ophthalmic preparations. Increasing resistance to antimicrobial agents is driving the search for safe and effective alternatives to traditional treatment methods. The aim of this study was to develop and evaluate gel-based ophthalmic formulations based on poloxamer 407 and sodium carboxymethylcellulose, containing propolis extracts and diclofenac sodium, by assessing their physical and chemical properties, antimicrobial activity, and cytotoxicity in vitro. Propolis extracts were prepared using a solvent consisting of ethanol and choline chloride, and the developed formulations were evaluated for pH, viscosity, refractive index, gelation temperature, SIRC cell viability and apoptosis, and antimicrobial activity against clinical and reference bacterial strains. The developed formulations exhibited physicochemical properties suitable for ophthalmic preparations and antimicrobial activity, particularly against Gram-positive bacteria. Most of the concentrations of the active ingredients tested were well tolerated by SIRC cells, although higher concentrations of the ethanol propolis extract caused a greater cytotoxic effect. The results obtained indicate that gelled ophthalmic formulations based on propolis extracts and diclofenac sodium are promising for the further development of topically acting ophthalmic preparations; however, their biological efficacy and safety must be confirmed. The results obtained indicate that gelled ophthalmic formulations based on propolis extracts and diclofenac sodium are promising for the further development of topical ophthalmic preparations; however, their bioavailability and safety must be confirmed by further in vivo studies.

## 1. Introduction

Vision is one of the most important senses in animals, ensuring environmental perception and information acquisition—up to 80% of external stimuli are received through the eyes [1]. In recent years, a significant global increase in the prevalence of ocular diseases, particularly inflammatory conditions, has been observed [2]. Inflammatory eye diseases and anterior segment disorders are among the most common ocular conditions in both veterinary and human medicine, largely due to the constant exposure of the eye to environmental factors [3,4]. Therefore, there remains a need for new therapeutic preparations, including natural substances, which could be used alone or in combination [5]. There has been a trend in recent decades for compounds of natural origin to receive significant attention as potential therapeutic agents in human and veterinary medicine [6]. The use of these materials has taken on new significance due to growing concerns about antimicrobial resistance (AMR)—one of the most serious challenges facing public health today [7,8,9]. Natural-origin products—particularly plant-based and bee-derived products—are valued for their biological activity, safety, and ecological compatibility. Natural compounds derived from plants, microorganisms, insects, or marine organisms exhibit structural and functional diversity, enabling them to act on a wide range of biological targets [10,11,12,13]. One of the most extensively studied bee products is propolis, which has a complex chemical composition and exhibits a broad spectrum of biological activity. The content of bioactive compounds in propolis varies significantly, as it is influenced by numerous factors, such as bee species, season, temperature, and geographical location. In this context, propolis represents a promising alternative due to its well-documented pharmacological properties, including anti-inflammatory, antioxidant, antimicrobial, antitumor, neuroprotective, and wound-healing effects [14,15,16,17]. Grecka et al. found that certain flavonoid fractions play a crucial role in the antibacterial activity of propolis [18]. These biological properties are attributed to the flavonoids (quercetin and pinocembrin), terpenes (bachcharin), and phenolic acids (artepilin C, *p*-coumaric acid, caffeic acid, and ferulic acid) found in propolis [19,20,21,22]. The results of our previous in vitro study with ethanolic propolis extracts demonstrated that the extracts did not inhibit bacteria belonging to the families *Enterococcaceae*, *Enterobacteriaceae*, and *Pseudomonadaceae*. However, the ethanolic propolis extracts were effective against *Staphylococcus aureus*, *Streptococcus agalactiae*, and *Bacillus cereus*. These findings are consistent with other scientific studies, which attribute the lower susceptibility of Gram-negative bacteria to their multilayered cell envelope structure and higher lipid content [23,24]. Substances such as phenolic compounds and flavonoids show great potential and form the basis for innovative therapeutic strategies that reduce the risk of drug resistance. Studies have shown that polyphenols can protect against the effects of UV radiation in various ways [25,26]. The application of phenolic compounds in ophthalmic eye drop formulations opens new opportunities for the treatment of glaucoma, dry eye syndrome, and other corneal injuries and inflammatory conditions [27,28]. The efficacy of ophthalmic preparations depends not only on the properties of the active ingredient, but also on the appropriate choice of dosage form, excipients, and their concentrations. Topically administered eye medications face numerous anatomical and physiological barriers, so their ability to penetrate deeper into the eye tissues is limited [29,30]. For these reasons, there is increasing interest in innovative drug delivery systems aimed at improving bioavailability and ensuring a prolonged therapeutic response. In recent years, polymer-based ocular drug delivery systems, particularly in situ gelling formulations, have been extensively investigated because of their ability to prolong precorneal residence time, improve ocular bioavailability, and enhance therapeutic efficacy. For these reasons, there is increasing interest in innovative ocular drug delivery systems aimed at improving ocular bioavailability and increasing precorneal residence time [31,32]. Poloxamer P407 and sodium carboxymethylcellulose (CMC Na) are often included to control viscosity and drug release kinetics, as they exhibit good biocompatibility and the ability to prolong the release of the active ingredient [33]. Since the eye is one of the most sensitive organs, new gelled ophthalmic preparations must be evaluated not only for their efficacy but also for their potential risk of irritation. In vitro cytotoxicity assays are widely used in modern pharmaceutical and veterinary practice, helping to reduce the need for animal testing. One of the most used methods employs the SIRC (Statens Seruminstitut Rabbit Cornea) rabbit corneal epithelial cell line, which allows for a rapid and reliable evaluation of the effect of preparations on corneal cell viability. The viability of SIRC (Statens Seruminstitut Rabbit Cornea) cells was assessed by flow cytometry, with the cells labeled using Annexin V/7-AAD dyes. This method allows for the differentiation of live cells from those in early and late apoptosis based on changes in phosphatidylserine distribution and membrane permeability.

Although numerous studies have examined the antimicrobial, antioxidant, and anti-inflammatory properties of propolis extracts, and ophthalmic drug delivery systems based on thermoreactive polymers are being extensively studied to improve the bioavailability of topically administered formulations, there is still insufficient data on gelled ophthalmic formulations intended for veterinary use in which propolis extracts are combined with diclofenac sodium. Furthermore, the literature lacks comparative studies evaluating the suitability of propolis extracts obtained using different extraction methods—including ethanol- and choline chloride-based solvents—for use in ophthalmic formulations, as well as their physicochemical properties, antimicrobial activity, and biocompatibility. The novelty of this study lies in the incorporation of a propolis extract based on choline chloride into thermoreactive ophthalmic gel formulations composed of poloxamer P407 and sodium carboxymethylcellulose. Although poloxamer-based in situ ophthalmic gels have been extensively studied, the use of propolis extracts obtained via a choline chloride-based aqueous solvent method in such systems remains limited. Therefore, the developed formulations represent an innovative approach that allows for the combination of propolis’s antimicrobial potential with the anti-inflammatory effect of diclofenac sodium in a thermoreactive ophthalmic drug delivery platform. In this study, diclofenac sodium was selected for its well-known pharmacological anti-inflammatory effects and wide application in ophthalmology, while propolis extracts were chosen for their antimicrobial potential. The combination of these active ingredients made it possible to develop a combined topical ophthalmic formulation designed to simultaneously address inflammation and bacterial infection, which frequently occur in eye diseases. Such a formulation may enable simultaneous targeting of different mechanisms of disease pathogenesis. Therefore, the aim of this study was to develop gel-based ophthalmic formulations based on poloxamer 407 and sodium carboxymethylcellulose, containing propolis extracts and diclofenac sodium, and to evaluate their physicochemical properties, antimicrobial activity, and in vitro cytotoxicity, in order to assess their potential as promising delivery systems for topically administered ophthalmic drugs in veterinary medicine.

This study will focus on the physical and chemical properties, antimicrobial activity, and cytotoxicity of the developed formulations. The results of this study pave the way for future research evaluating inflammatory biomarkers and therapeutic efficacy in vivo, with the aim of determining whether the combination of diclofenac sodium and propolis extracts produce an additive or synergistic anti-inflammatory effect.

## 2. Results and Discussion

### 2.1. Characterization of Propolis Extract

This study evaluated the potential of choline chloride as a solvent for propolis extraction. The total phenolic content was determined in a 10% solution. The study found that the total content of phenolic compounds in the propolis extract prepared with choline chloride was 102 mg/g ± 0.44 GAE (Gallic acid equivalents). This confirms the findings of studies conducted by other researchers in which organic acids were used for propolis extraction and the total phenolic compound content ranged from 17.3 mg/g GAE to 136.2 mg/g GAE [34]. The authors investigated various NADES systems and demonstrated that choline chloride-based solvents can effectively extract propolis polyphenols while maintaining high biological activity [35]. The total content of phenolic compounds was also determined in the ethanol propolis extract, where the content of phenolic compounds was 127 mg/g ± 2 GAE. The results of our previous study showed that a similar number of phenolic compounds was found in the ethanol extract of propolis [36]. Future studies should employ FTIR, HPLC, or additional spectrophotometric methods to determine the complete chemical composition, confirm chemical compatibility, and rule out undesirable interactions between the components of the formulated products prior to further pharmaceutical development.

### 2.2. Compositions and Physicochemical Properties of Experimental Formulations

**Figure 1:**
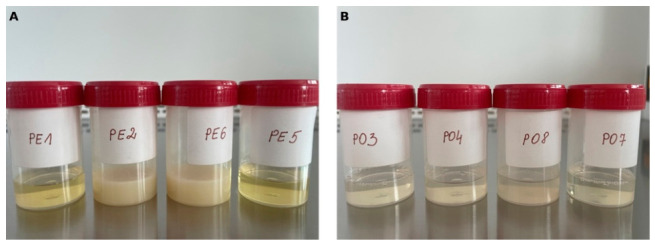
Representative images of (**A**) PE mixtures (PE1, PE2, PE6, PE5) and (**B**) PO mixtures (PO3, PO4, PO8, PO7). Differences in visual appearance, including color intensity and turbidity, are evident between the formulations.

All the ophthalmic formulations developed were visually homogeneous, but slight differences in appearance were observed among them. The appearance of the formulations differed between the PE and PO groups. The PE ophthalmic formulations (PE1, PE2, PE6, PE5) (Figure 1A) exhibited greater visual differences. The PE1 and PE5 ophthalmic formulations were relatively transparent and light yellow in color, whereas the PE2 and PE6 ophthalmic formulations were opaquer and more had a whitish tint. Color differences between formulations based on ethanol propolis extract and carboxymethylcellulose may be related to changes in the solubility of propolis phenolic compounds in an aqueous polymeric medium, their interaction with sodium carboxymethylcellulose, and the formation of fine dispersed particles. For these reasons, light scattering and optical properties change, so the formulations may appear cloudier or have a whitish tint [37,38]. The ophthalmic formulations (PO3, PO4, PO8, PO7) (Figure 1B) were visually more homogeneous. All samples were clear and light yellow in color.

When evaluating the physicochemical parameters of ophthalmic formulations after manufacture, the pH values at 22 ± 1 °C were close to those of natural tears, as were the values 14 days after manufacture when stored at 5 ± 1 °C. (Table 1) The PE formulations (PE1, PE2, PE5, PE6) were characterized by slightly higher pH values (7.06 ± 0.11–7.19 ± 0.03), whereas the PO formulations (PO3, PO4, PO7, PO8) had lower pH values (6.43 ± 0.29–6.50 ± 0.03). After 14 days, the PE formulations (PE1, PE2, PE5, PE6) exhibited slightly higher pH values (7.03 ± 0.10–7.16 ± 0.03), whereas the PO formulations (PO3, PO4, PO7, PO8) had lower pH values (6.40 ± 0.28–6.48 ± 0.03).

The slightly higher pH of the PE formulations may be related to the composition of the ethanol propolis extract, which may contain fewer acids. Meanwhile, the lower pH of the PO formulations may be associated with the propolis extract prepared on a choline chloride basis, which may contain more acidic functional groups or exhibit stronger hydrogen bonding interactions with the solvent. To ensure maximum eye comfort, ophthalmic formulations should have a pH close to neutral (7.2) [39]. However, previous studies show that the pH values of ophthalmic formulations can range from 3.5 to 8.5 [40]. The pH of all formulations was within the acceptable range, and it can be concluded that the extract does not adversely affect the pH values of the formulations [41,42].

The refractive index values of the ophthalmic formulations at 22 ± 1 °C after production were very similar and ranged from 1.337 to 1.341. Fourteen days after manufacture, when the formulations were stored at 5 ± 1 °C, the refractive index values ranged from 1.337 to 1.340 (Table 1) [43]. It is recommended that the refractive index of eye care products not exceed 1.476 [44]. The refractive index results show that all ophthalmic formulations are optically similar and have similar solution density and composition. After storage in the refrigerator, their appearance, pH, and refractive index remained unchanged, indicating that no phase separation or degradation of the substances occurred in the gel formulations. A slight increase in the refractive index in PO formulations may be related to a higher concentration of dissolved substances or stronger intermolecular interactions characteristic of solvents such as mixtures based on choline chloride.

**Figure 2:**
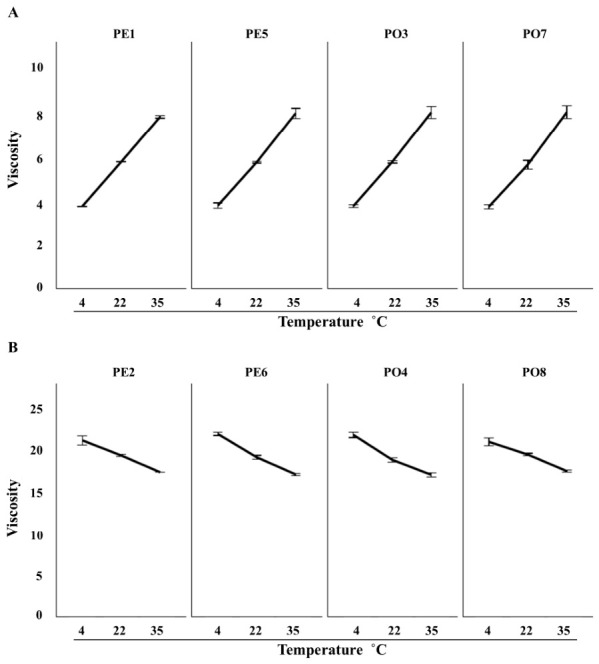
Temperature-dependent changes in viscosity (mPa·s) of (**A**) (PE1, PO3, PE5, PO7) and (**B**) mixtures (PE2, PO4, PE6, PO8). Data are presented as mean ± standard deviation.

The ophthalmic formulations were divided into two groups based on their viscosity results: Group A formulations: (PE1, PO3, PE5, PO7). The viscosity of all formulations containing poloxamer P407 increased with rising temperature from 4 ± 1 °C to 35 ± 1 °C. At a temperature of 4 ± 1 °C, the viscosity was 3.68 ± 0.08–3.74 ± 0.1 mPa·s; at 22 ± 1 °C, the viscosity of the formulations was 5.57 ± 0.16–5.70 ± 0.04 mPa·s, and at 35 °C, it reached 7.74 ± 0.04–7.95 ± 0.2 mPa·s (Figure 2A). Group B formulations: (PE2, PO4, PE6, PO8). In all formulations containing sodium carboxymethylcellulose, the opposite trend was observed viscosity decreased as temperature increased. At 4 ± 1 °C, viscosity was highest (20.82 ± 0.8–21.76 ± 0.3 mPa·s), at 22 ± 1 °C, the viscosity of the formulations was 18.68 ± 0.4–19.30 ± 0.2 mPa·s, and at 35 ± 1 °C, it decreased to 16.90 ± 0.4–17.34 ± 0.2 mPa·s (Figure 2B).

Polymer concentration is one of the most important factors determining the properties of thermoreactive ophthalmic gels. As the concentration of poloxamer 407 (P407) increases, the sol–gel transition temperature decreases and viscosity increases due to more intense micellization and a denser micelle distribution. These properties allow the formulation to remain liquid upon instillation and to gel rapidly upon reaching the temperature of the ocular surface (32–35 °C), thereby prolonging contact time and ensuring controlled release of the active ingredients [45]. Sodium carboxymethylcellulose (NaCMC) increases viscosity and mucoadhesion, thereby prolonging the time the preparation remains on the surface of the eye. This property is characteristic of cellulose-derived polymers and is associated with increased mobility of polymer chains at higher temperatures, which weakens intermolecular hydrogen bonds and reduces chain entanglement. Consequently, the flow resistance of the polymer network decreases, resulting in lower viscosity [46]. P407 is one of the most extensively studied thermoreactive polymers in ophthalmic drug delivery systems due to its good biocompatibility and safety. In vitro, ex vivo, and in vivo studies have shown that P407 does not cause significant corneal irritation or cytotoxic effects, making it suitable for topical ophthalmic formulations [47,48]. Although high (≥20%) concentrations of P407 administered intravitreally may be associated with adverse effects on the retina [49], such an effect is not characteristic of preparations intended for the anterior segment of the eye. The concentrations of 7% P407 and 0.25% NaCMC used in this study ensured an appropriate gelation temperature, physiological viscosity, and transparency, confirming their suitability for thermoreactive gel ophthalmic formulations.

Gel-forming polymers protect the surface of the eye, improve the distribution of medications, and prolong their contact with the tissues, thereby increasing the absorption of the active ingredients [50]. Formulations based on Poloxamer P407 are temperature-sensitive—as the temperature rises, micelles and a three-dimensional network form, resulting in increased viscosity. In contrast, the viscosity of NaCMC-based systems decreases as the temperature rises due to weakened interactions between the polymer chains and the solvent [51]. The results showed that the viscosity of all the ophthalmic formulations tested fell within the range tolerated by the eye and can ensure proper distribution on the ocular surface. When selecting an extract for ophthalmic use, safety for the eyes and physicochemical compatibility should also be considered. Although the osmolarity of the formulations developed in this study was not determined, this parameter is important for assessing the tolerability of ophthalmic preparations. Sebbag et al. found that the osmolarity of tears in healthy dogs is 337.4 ± 16.2 mOsm/L; therefore, the osmolarity of ophthalmic preparations should be as close as possible to the physiological values of tear fluid in order to reduce eye irritation, reflex tearing, and ensure longer contact of the formulation with the ocular surface. As the quality assessment of ophthalmic formulations is expanded in the future, it would be advisable to conduct studies on ocular tolerance to osmolarity [52,53].

### 2.3. Evaluation of the Antibacterial Activity of Ophthalmic Formulations

An in vitro assessment of antimicrobial activity revealed that ethanol-based gel propolis formulations (PE) exhibited the strongest activity against Gram-positive bacteria, particularly strains of *Staphylococcus aureus* and *Bacillus cereus*, for which the largest zones of growth inhibition were observed. Choline chloride-based gel propolis formulations (PO) demonstrated moderate antibacterial activity against all tested bacteria, including *Enterococcus faecalis*, *Escherichia coli*, and *Pseudomonas aeruginosa*, against which the PE formulations were ineffective. The positive control formulation PO0 exhibited antibacterial activity against all tested bacterial strains. Although no statistically significant differences were found between PO0 and the propolis-containing formulations, the latter showed a tendency toward greater antibacterial activity, suggesting that the inclusion of propolis in gel formulations may enhance their antimicrobial effect. The negative ethanol (70% ethanol) control group did not exhibit any antibacterial activity against any of the bacterial strains tested. Diclofenac sodium 0.25% salt exhibited antibacterial activity only against Gram-positive bacteria but was ineffective against Gram-negative bacteria and *Enterococcus faecalis*. The results indicate that the antibacterial activity of propolis formulations depended on the solvent system used and the bacterial species (Table 2). The more pronounced antibacterial activity of propolis extracts against Gram-positive bacteria can be explained by the multifactorial mechanism of action of phenolic compounds and flavonoids. These compounds disrupt the integrity of the bacterial cytoplasmic membrane, increase its permeability, inhibit enzyme activity, protein synthesis, and nucleic acid replication, and interfere with bacterial adhesion and biofilm formation. In addition, some phenolic compounds can promote the formation of reactive oxygen species (ROS) in bacterial cells, causing oxidative damage to membranes, proteins, and DNA; as a result, it is more difficult for bacteria to develop resistance to propolis than to conventional agents [54]. In addition, Gram-positive bacteria have a thick peptidoglycan layer but lack an outer membrane, so phenolic compounds and flavonoids can more easily penetrate the cell and damage the cytoplasmic membrane. In contrast, Gram-negative bacteria possess an additional outer membrane rich in lipopolysaccharides (LPS), which form an effective barrier against hydrophobic compounds, including many of the phenolic components of propolis. Furthermore, Gram-negative bacteria are more likely to possess active efflux systems and have lower membrane permeability, so their sensitivity to propolis extracts is generally lower [16,55]. In our study, the ethanol extract of propolis exhibited greater antibacterial activity than the choline chloride extract, likely due to its higher total phenolic content and higher concentrations of major phenolic acids. This confirms that the chemical composition of propolis is one of the most important factors determining its biological activity.

**Figure 3:**
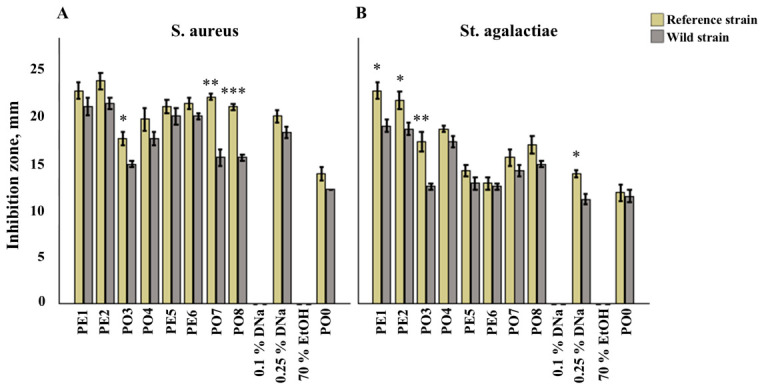
Antibacterial activity against *S. aureus* (**A**) and *St. agalactiae* (**B**). The asterisks indicate statistical significance between bacterial strains and formulations * *p* < 0.05, ** *p* < 0.01, *** *p* < 0.001.

Research by Polish scientists has shown that propolis exhibits strong antibacterial activity against various Gram-positive and Gram-negative bacteria, including *S. aureus*, *E. coli*, and *Ps. aeruginosa*. Due to its broad spectrum of activity, propolis is considered a promising natural antimicrobial agent, particularly important in the fight against antibiotic-resistant bacteria [56]. The data presented showed that the ophthalmic formulations tested exhibited varying degrees of antibacterial activity against *S. aureus* (Figure 3A) and St. agalactiae (Figure 3B) strains. When evaluating the inhibition of *S. aureus* growth, a statistically significantly greater antibacterial effect was observed for formulations PO7 (** *p* < 0.01), PO8 (*** *p* < 0.001), and PO3 (* *p* < 0.05). Meanwhile, statistically significant growth inhibition against *St. agalactiae* was observed for the PE1 (* *p* < 0.05), PE2 (* *p* < 0.05), PO3 (** *p* < 0.01), and 0.25% DNa (* *p* < 0.05) formulations. These results indicate that different propolis formulations inhibit the growth of Gram-positive bacteria with varying degrees of effectiveness. Scientific studies show that clinical *S. aureus* strains often exhibit greater resistance to antimicrobial agents than reference strains, which is why smaller zones of growth inhibition are observed in the case of wild-type strains. Recent studies also confirm that both ethanol-based and natural solvents effectively inhibit the growth of *S. aureus* [16,57,58].

The literature indicates that the mechanisms of action of organic acid mixtures are not yet fully understood, but studies show that they have a significant effect in reducing *Streptococcus agalactiae* infection. It has been found that these mixtures inhibit the expression of bacterial capsule polysaccharides, reduce inflammatory responses, and can be used as a preventive measure in aquaculture [59].

**Figure 4:**
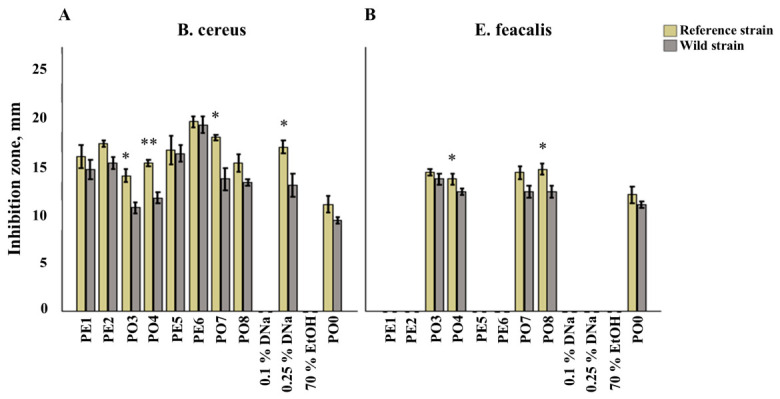
Antibacterial activity against *B. cereus* (**A**) and *E. faecalis* (**B**). The asterisks indicate statistical significance between bacterial strains and formulations * *p* < 0.05, ** *p* < 0.01.

The results presented showed that the ophthalmic formulations tested exhibited antibacterial activity against B. cereus (Figure 4A). When evaluating the inhibition of *B. cereus* growth, a statistically significant difference between the reference strain and the wild-type strain was observed for the ophthalmic formulations PO4 (** *p* < 0.01), PO3 and PO7 (* *p* < 0.05), and 0.25% DNa (* *p* < 0.05). Meanwhile, in the case of *E. faecalis,* the overall antibacterial effect was lower, but statistically significant growth inhibition was observed for formulations PO4 (* *p* < 0.05) and PO8 (* *p* < 0.05) (Figure 4B). The results indicate that different propolis formulations inhibited the growth of Gram-positive bacteria with varying degrees of effectiveness, with a stronger effect observed against *B. cereus* strains. Our study, which examined gel-based propolis systems against strains of several bacterial species, including *B. cereus*, demonstrated that formulations containing natural propolis extracts exhibited significant antibacterial activity against *B. cereus* [36]. Solvent PO0 exhibited limited antibacterial activity; however, this activity is not sufficient to be considered equivalent to that of the active formulations and merely complements their antibacterial activity. This confirms that the observed effect of the active formulations is not solely attributable to the solvent. According to data from Polish researchers, propolis exhibits strong antibacterial activity against Gram-positive and Gram-negative bacteria, including *S. aureus*, *Bacillus* spp., *E. faecalis*, *E. coli*, and *Ps. aeruginosa*. It has been found that Gram-positive bacteria are generally more sensitive to the phenolic compounds in propolis due to their simpler cell wall structure [60]. Meanwhile, studies of Nepalese propolis have shown similar antibacterial activity against both Gram-positive and Gram-negative bacteria, which is attributed to differences in the composition of bioactive compounds [61]. Similarly, propolis from the Middle East demonstrated strong activity against both *S. aureus* and *E. coli*, highlighting that regional plant-based sources may confer a broader spectrum of antimicrobial activity [16]. During the study, we observed that diclofenac sodium exhibited antibacterial activity only at concentrations of 0.25% or higher. In the study, diclofenac inhibited *S. aureus*, *St. agalactiae*, and *B. cereus* strains. Nonsteroidal anti-inflammatory drugs (NSAIDs) have analgesic, anti-inflammatory, and antipyretic effects. Recently, in some studies, diclofenac sodium has been recognized as having antimicrobial activity, like many non-antibiotic drugs [62]. Diclofenac exhibits in vitro antimicrobial activity against various bacterial strains, including *E. coli*, *S. aureus*, and *B. subtilis*. Ibuprofen and diclofenac are also active against *E. faecalis*, although their activity is weaker than that of classic antibiotics such as amoxicillin or gentamicin [63]. It is believed that the antibacterial mechanism of diclofenac is primarily related to the inhibition of DNA synthesis. Data suggest that NSAIDs may act not only as anti-inflammatory agents but also as adjuvants in the treatment of infectious diseases, by modulating bacterial virulence or enhancing the efficacy of antimicrobial therapy [64].

**Figure 5:**
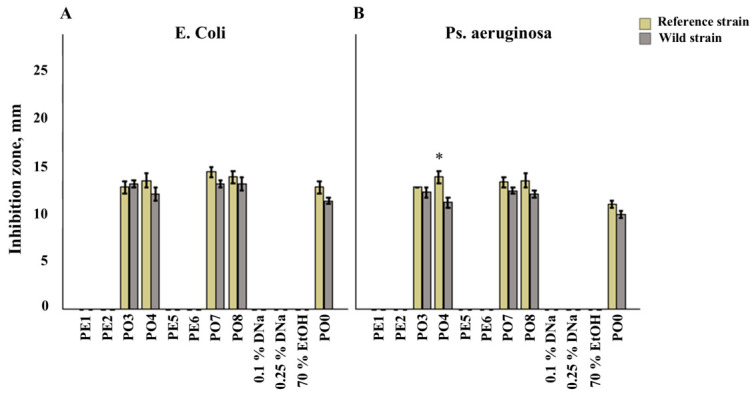
Antibacterial activity against *E. coli* (**A**) and *Ps. Aeruginosa* (**B**). The asterisks indicate statistical significance between bacterial strains and formulations * *p* < 0.05.

When evaluating the inhibition of *E. coli* growth, formulations containing choline chloride as a solvent exhibited antibacterial activity (Figure 5A). The largest inhibition zones were observed with formulations PO7 and PO8; however, no statistically significant differences were found between the strains. Meanwhile, against *Ps. aeruginosa*, all active ophthalmic formulations caused moderate growth inhibition, and a statistically significant difference was observed for the PO4 ophthalmic formulation (* *p* < 0.05), (Figure 5B). The overall antibacterial effect against Gram-negative bacteria was weaker than against Gram-positive strains, which may be associated with the more complex structure of the outer membrane of Gram-negative bacteria.

These results are consistent with recent studies showing that natural solvents can increase the extraction efficiency and antibacterial activity of phenolic compounds compared to traditional ethanol extracts [65]. Studies have also shown that propolis extracts based on choline chloride may be a promising alternative to hydroethanolic extracts, especially in cases where ethanol must be avoided. It was found that a mixture of choline chloride and 1,2-propanediol exhibited the highest extraction efficiency and biological activity [66]. The literature indicates that natural eutectic propolis extracts exhibit a stronger effect against both Gram-positive and Gram-negative bacteria, including *E. faecalis*, *E. coli*, and *Ps. aeruginosa*, compared to traditional ethanol extracts [67,68]. In addition, the results of our recent study showed that organic propolis extracts made from raw propolis sourced from different countries (Lithuania, Latvia, Poland) effectively inhibited the growth of clinical and reference strains of *S. aureus*, *St. agalactiae*, *B. cereus*, *E. faecalis*, *E. coli*, and *Ps. Aeruginosa* [23]. Based on the physical and chemical properties of thermoactivated gels and on well-known data from the literature regarding the characteristics of in situ gels made from poloxamer, it is expected that these formulations will increase the duration of drug retention in the eye [69]. It has also been found that the micellar structure of poloxamers improves the stability of hydrophobic phenolic compounds [70]. The results of the study confirmed that carboxymethylcellulose (CMC) significantly accelerates corneal epithelial healing and promotes complete epithelialization [71]. In summary, it can be stated that combinations of poloxamers and cellulose derivatives constitute a promising system for enhancing the stability, bioavailability, and antibacterial activity of propolis’s active compounds, and may improve tissue regeneration processes.

### 2.4. Evaluation of SIRC Cell Viability by Flow Cytometry

Cell lines are a suitable biological system for evaluating the toxicity and biological activity of new substances or formulations. In this study, the cytotoxicity of ophthalmic formulations was evaluated using the SIRC (ATCC CCL-60) cell line, which is widely used for the preliminary assessment of the biocompatibility of ophthalmic preparations. This model allows for the assessment of the direct effects of the preparations on the viability and apoptosis of corneal epithelial cells, making it suitable for the early safety evaluation of ophthalmic preparations. The results of this study should be considered a preliminary assessment of biological safety, and the clinical applicability of the developed formulations should be further evaluated in ex vivo and in vivo models.

In this step, different concentrations of the active substances were selected to assess their effects on SIRC cell viability and apoptosis, considering real-world ophthalmic conditions. The selected concentrations reflect the potential dilution of the formulation on the ocular surface after instillation, as the ophthalmic formulation (~40 µL) mixes with tear fluid and is diluted approximately 1–3 times [53,72]. The time intervals of 15 min, 2 h, and 4 h were selected based on the pharmacokinetics of ophthalmic preparations and the actual conditions of contact with the ocular surface, since most instilled substances are eliminated within a few minutes, and a significant concentration on the ocular surface persists for a limited time, resulting in limited long-term exposure under natural conditions. The contact time of ophthalmic preparations with the ocular surface often does not exceed 2–5 min, and the biological effect depends on the early phase of exposure; therefore, short time intervals are considered appropriate for assessing the initial cytotoxic effect. Additional longer time points (2 and 4 h) were included in this study to assess potential delayed effects and to provide a more conservative safety assessment under in vitro conditions, where natural clearance mechanisms are absent. Based on these data, a concentration range was selected for in vitro studies that included both lower (diluted) and higher (close to the original formulation) concentrations. This selection allows for a better assessment of both safety margins and potential cytotoxic effects under real-world conditions, as well as the determination of the concentration at which the formulations remain biologically safe for corneal epithelial cells. The cytotoxic activity of propolis has been extensively studied, particularly in the field of cancer research [73,74]. Various propolis extracts have been found to exhibit strong cytotoxic effects on different human tumor cell lines. In their study, Silva and colleagues evaluated the effects of ethanol extracts of Brazilian red propolis on OVCAR-8, HCT-116, HL-60, and SF-295 cells, and demonstrated marked antiproliferative activity. Similar conclusions were presented by Watanabe and his research team after reviewing the anticancer effects of propolis components [75,76]. However, there is little data on the cytotoxic effects of ophthalmic formulations containing propolis on the SIRC cell line. Studies described in the literature that evaluated the effects of ophthalmic formulations containing poloxamer P407 and hydroxypropyl methylcellulose on corneal epithelial fibroblasts showed that neither the polymers nor the active ingredient caused any cytotoxic effects [77].

**Figure 6:**
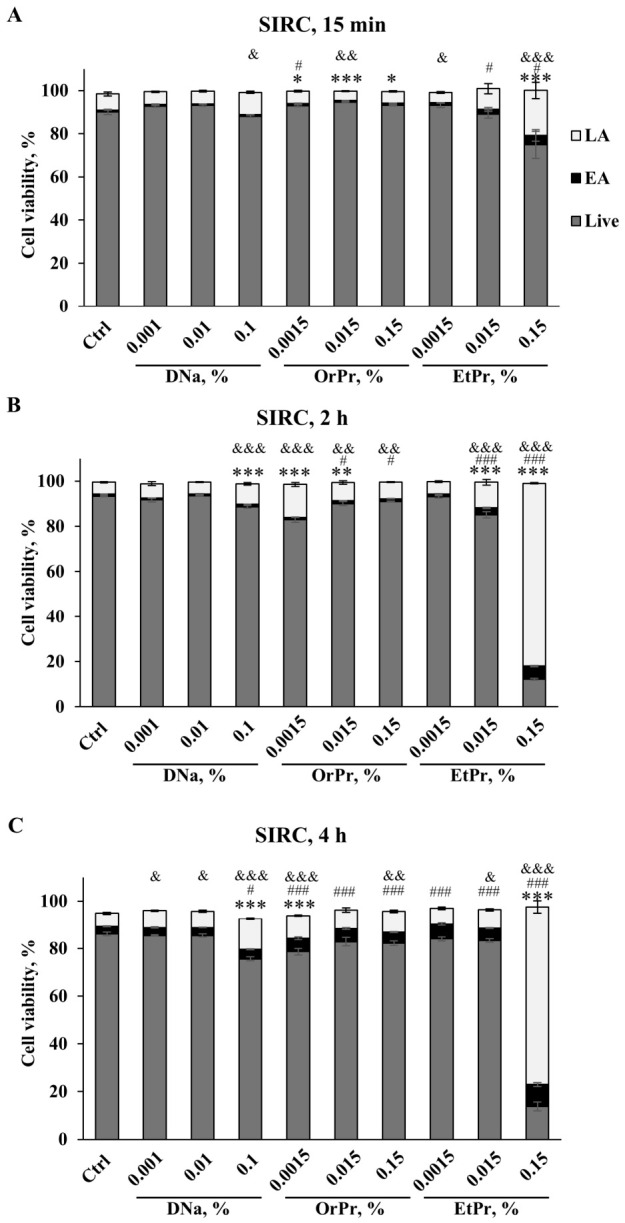
Effect of the test compounds on SIRC cell viability and apoptosis. The effect of DNa, OrPr and EtPr on SIRC cell viability was evaluated after 15 min (**A**), 2 h (**B**) and 4 h (**C**). Data are presented as mean ± standard deviation error. *^, #, &^ *p* < 0.05, **^, &&^ *p* < 0.01, ***^, ###, &&&^ *p* < 0.001. *—for live cells, ^#^—for EA (early apoptosis), ^&^—for LA (late apoptosis).

Assessment of SIRC cell line viability and early (EA) and late (LA) apoptosis after 15 min of exposure to the test substances showed varying results depending on the type and concentration of the substance. At all tested concentrations of diclofenac sodium (DNa) (0.001%, 0.01%, and 0.1%), cell viability remained high (92.68%, 93.19%, and 88.12%), while a significant difference in the late apoptosis cell group was observed only with 0.1% DNa. Upon treatment of cells with organic propolis extract (OrPr), increased cell viability was observed at all OrPr concentrations (0.0015–0.15%), which may be related to its antioxidant and cytoprotective properties [75]. The literature also indicates that propolis extracts may maintain or even improve cell viability under in vitro conditions, particularly at lower concentrations or in the presence of oxidative stress, by preserving viability and promoting cell proliferation [78,79]. A significant decrease in SIRC cell viability and an increased number of apoptotic cells were observed following treatment with an ethanol propolis extract (EtPr). After treatment with 0.0015% and 0.015% EtPr, SIRC cell viability remained high (93.18% and 89.11%), but a significant decrease in viability was observed at 0.15% ErPr (to 74.83%, *** *p* < 0.001), along with an increased number of cells undergoing both early (4.39%) and late apoptosis (20.84%) (Figure 6A). An experiment was conducted using the CCF-STTG1 astrocyte cell line, and the study showed that propolis extracts can induce early and late apoptosis by affecting the cell cycle and mitochondrial function, and this effect is particularly pronounced at higher concentrations [80].

After conducting SIRC cell viability tests following a 2 h exposure, we found that 0.001% and 0.01% DNa had no effect on cell viability, while 0.1% DNa reduced cell viability from 93.37% to 88.64%. We found that 0.0015% and 0.015% OrPr reduced SIRC cell viability to 89.02% and 90.08%, respectively, while 0.15% OrPr did not alter the number of viable cells but did alter the ratio of early- and late-stage apoptotic cells. Meanwhile, 0.0015% EtPr did not cause changes in SIRC cell viability, but at higher concentrations (0.015% and 0.15%) it reduced the viability of these cells (to 85.12% and 12.21%, respectively) (Figure 6B). Researchers investigated the mechanisms of propolis-induced apoptosis, and it appears that apoptosis depends on the type of compounds and the concentration of the propolis extract. Following in vitro studies, they determined that cancer cells exhibit varying sensitivity to propolis and its compounds [81]. Furthermore, recent studies confirm that the phenolic compounds in propolis can lead to the formation of reactive oxygen species (ROS), which trigger oxidative stress and activate apoptotic signaling pathways [82]. Chilean researchers also investigated the protective effects of propolis extract on pancreatic β-cells during oxidative stress in vitro and observed that propolis extracts exert a significant protective effect on β-cells exposed to oxidative stress [83].

After incubating SIRC cells with the test substances for 4 h, we found that 0.001% and 0.01% DNa did not affect the number of viable cells, whereas 0.1% DNa reduced cell viability from 86.04% to 75.63%. Studies indicate that diclofenac sodium may have a significant effect on corneal epithelial cells. When investigating the cytotoxic activity of diclofenac in the RCEC cell line, cell viability decreased as the concentration of the active substance and the duration of incubation increased. At the same time, flow cytometry analysis revealed cell cycle arrest in the G0/G1 phase and a marked increase in apoptosis, indicating that diclofenac not only inhibits proliferation but also initiates programmed cell death. These results suggest that diclofenac sodium exhibits a dose-dependent inhibitory effect on corneal epithelial cells and acts through mechanisms of apoptosis and cell cycle regulation [84]. The cytotoxicity of 0.1% diclofenac sodium was also confirmed by Brazilian researchers who studied alkali-induced burns in the corneas of healthy rabbits; their data showed that the effect of NSAID eye drops on the corneal epithelium depends on the type and concentration of the active ingredient, and the ketorolac tromethamine formulation is associated with a higher risk of cytotoxic effects [85]. OrPr (0.015% and 0.15%) did not cause changes in viable cells, but a higher number of apoptotic cells was observed at these concentrations. The lowest OrPr concentration (0.0015%) reduced SIRC cell viability from 86.04% to 78.66% and caused an increase in the number of cells undergoing early and late apoptosis. At low concentrations (0.0015% and 0.015%), EtPr maintained high cell viability (84.01% and 83.35%) and did not cause statistically significant changes, but a higher number of apoptotic cells was observed. Meanwhile, the use of 0.15% EtPr resulted in a marked decrease in cell viability to 13.80% and a significant increase in the number of cells undergoing early (9.18%) and, particularly, late apoptosis (74.60%) (Figure 6C). The study found that propolis extracts at lower concentrations may be biologically compatible with ocular tissues, but at higher concentrations may induce cell apoptosis. Recent in vivo studies by Brazilian researchers using rats showed that the ocular surface tolerated well the instillation of ophthalmic preparations containing standardized green propolis extract at concentrations of 5, 10, and 15 mg/mL every 6 h for 4 consecutive days. Despite their evident anti-inflammatory and anti-angiogenic effects, the propolis formulations did not exhibit hepatotoxic, nephrotoxic, or genotoxic effects, which supports their use in the development of new ophthalmic preparations [86]. This study found that higher concentrations of propolis extract reduced the viability of SIRC cells, suggesting that the biological activity of propolis is concentration dependent. Although the phenolic compounds in propolis exhibit antioxidant and cytoprotective properties at low concentrations, at higher concentrations they can disrupt the cellular redox balance, increase the formation of reactive oxygen species (ROS), impair mitochondrial function, and activate apoptotic signaling pathways. Furthermore, high concentrations of phenolic compounds can alter the permeability and integrity of cell membranes, thereby reducing cell viability [81,87].

These results indicate that, when developing ophthalmic propolis formulations, it is necessary to optimize the concentration of propolis extract to maintain sufficient antimicrobial activity while ensuring good biocompatibility with corneal epithelial cells. To provide a comprehensive interpretation of the results, both antibacterial activity and compatibility with SIRC cells were considered when evaluating the developed formulations. Formulations containing organic propolis extract (PO group) exhibited the most favorable balance: they exhibited moderate antibacterial activity against all tested bacterial strains while maintaining high SIRC cell viability and cytocompatibility, indicating a favorable safety profile.

This study focused primarily on the physical and chemical properties, antimicrobial activity, and in vitro cytocompatibility of the developed ophthalmic formulations; their future pharmaceutical development requires consideration of manufacturing-related aspects: aseptic manufacturing methods compatible with the formulation’s components should be implemented before the developed formulations are introduced into clinical use.

## 3. Conclusions

The ophthalmic formulations, gelled based on poloxamer P407 (7%, *w*/*v*) and sodium carboxymethylcellulose (0.25%, *w*/*v*), exhibited suitable physicochemical properties. The pH, viscosity, and refractive index values of all formulations met the requirements for topical ophthalmic preparations.

The ethanol propolis extract formulations (PE) exhibited the highest antibacterial activity against the tested Gram-positive bacteria (*Staphylococcus aureus*, *Streptococcus agalactiae*, and *Bacillus cereus*), but were ineffective against *Enterococcus faecalis*, *Escherichia coli*, and *Pseudomonas aeruginosa*. Meanwhile, propolis extract formulations (PO) based on choline chloride exhibited a broader spectrum of antibacterial activity and inhibited the growth of all tested bacteria, although their activity against Gram-positive bacteria was lower than that of the PE formulations.

During short-term exposure, all formulations exhibited good biocompatibility with SIRC cells; however, higher concentrations of active components were associated with a greater risk of cytotoxicity. Given the results regarding the spectrum of antibacterial activity and biocompatibility, PO3 and PO4 are considered the most promising for further preclinical studies. Prior to their clinical application, it is necessary to conduct studies on the release, stability, sterility, in vivo efficacy, and long-term biological safety of the active ingredients.

## 4. Materials and Methods

### 4.1. Materials

Raw Lithuanian propolis was obtained from the apiary “Bičių korys” (Vilnius, Lithuania). Choline chloride and sodium carboxymethylcellulose (CMC Na) were purchased from Sigma-Aldrich (St. Louis, MO, USA), poloxamer 407 (P407) was supplied by Fagron (St. Paul, MN, USA), and diclofenac sodium was obtained from MedChemExpress (Monmouth Junction, NJ, USA). Purified water and 96.3% food-grade rectified ethanol (UAB “Vilniaus degtinė”, Vilnius, Lithuania) were used for formulation preparation. The formulations were prepared using laboratory electronic balances (Kern PBS/PBJ, KERN & SOHN GmbH, Balingen, Germany), an analytical balance (Scaltec SBC 31, Scaltec Instruments GmbH, Göttingen, Germany), a pH meter (Model 766) equipped with a Knick SE 104 N electrode (Knick Elektronische Messgeräte GmbH & Co., Berlin, Germany), and a magnetic stirrer with a heating plate (IKAMAG C-MAG HS7, IKA-Werke GmbH & Co. KG, Staufen im Breisgau, Germany). The SIRC rabbit corneal cell line (ATCC CCL-60; American Type Culture Collection, Manassas, VA, USA) was used for the in vitro cytotoxicity studies.

### 4.2. Extraction and Formulation Preparation Methods

#### 4.2.1. Preparation of Ethanolic Propolis Extract

The extraction procedure was adapted from the method described by Huang at al. [88]. The ground propolis was extracted using 70% ethanol (*v*/*v*) via the maceration method. The ratio of propolis to solvent was 1:10. The macerated mixture was stored in dark glass bottles at a temperature of 21 ± 1 °C for 12 days. During this period, the extract was stirred three times, for 30 min each time, on a magnetic stirrer, without heating. After maceration, the extract was stored in a refrigerator for 24 h. The extract was then filtered through ashless filter paper. The filtered extract was stored in dark glass bottles.

#### 4.2.2. Preparation of Propolis Extract Based on Choline Chloride

The extraction procedure was adapted from the method described by Maugeri and de María [89]. A choline chloride-based solvent (PO0) was prepared separately by adding the same amounts of the ingredients as specified above and stirring at a specific temperature. Choline chloride and purified water were mixed in a 1:1 ratio. The crushed propolis was extracted with the choline chloride solvent using the maceration method at a ratio of 1:10. The macerated mixture was stored in dark glass bottles (21 ± 1 °C) for 12 days. During this period, the extract was stirred three times, for 30 min each, on a magnetic stirrer, without heating. After maceration, the extract was stored in a refrigerator for 24 h. The extract was then filtered through ashless filter paper. The filtered extract was stored in dark glass bottles.

#### 4.2.3. Determination of Total Phenolic Content

The method is based on a colorimetric oxidation/reduction reaction using the Folin–Ciocalteu reagent. The method used is based on the general procedure recommended by the European Pharmacopoeia, with minor modifications. 10 µL of each extract was diluted with 1590 µL of purified water and mixed with 100 µL of Folin–Ciocalteu reagent for 6 min, after which 300 µL of a 20% sodium carbonate solution was added [90]. The mixture was incubated for 2 h at room temperature. The absorbance of the solutions was measured at a wavelength of 760 nm using an Agilent Technologies 8453 UV-Visible Spectrophotometer (Santa Clara, CA, USA). The total phenolic content was expressed in milligrams of gallic acid equivalents (GAE) per milliliter of extract.

#### 4.2.4. Formulation of Ophthalmic Preparations

Ophthalmic formulations were prepared using 7% (*w*/*v*) Poloxamer 407 (P407) or 0.25% (*w*/*v*) sodium carboxymethylcellulose (Na CMC) as gelling polymers. The formulations contained 0.1% or 0.25% (*w*/*v*) diclofenac sodium, 10% (*v*/*v*) ethanolic propolis extract or 10% (*v*/*v*) choline chloride-based propolis extract, and purified water to a final volume of 100 mL (Table 3).

To prepare the P407 formulations, 7 g of Poloxamer 407 was dispersed in purified water and stored at 5 °C for 24 h until complete hydration. The Na CMC formulations were prepared by dispersing 0.25 g of sodium carboxymethylcellulose in purified water under continuous stirring at 50 °C until a homogeneous gel was obtained. The polymer gels were then mixed using a magnetic stirrer. Diclofenac sodium powder was accurately weighed and dissolved in purified water to prepare stock solutions. The calculated volume of each stock solution was added under continuous stirring to obtain final diclofenac sodium concentrations of 0.1% (*w*/*v*) or 0.25% (*w*/*v*). Subsequently, 10 mL of either ethanolic propolis extract or choline chloride-based propolis extract was incorporated into each formulation and mixed until homogeneous. The prepared formulations were stored at 5 °C until further analysis.

#### 4.2.5. Sterilization of Ophthalmic Formulations

The formulations were prepared in a vertical laminar flow cabinet (Airstream^®^ Gen 3, Esco Life Sciences, Singapore). The prepared eye drops were filtered through a sterile 0.22 µm membrane filter and then aseptically filled into sterile eye drop vials. This method was chosen to avoid potential thermal degradation of diclofenac sodium and heat-induced changes in the polymeric composition of the ophthalmic preparation.

### 4.3. Characterization Methods

#### 4.3.1. pH of Ophthalmic Gels

The pH of the formulated experimental hydrogels was measured at room temperature (22 ± 1 °C) using a pH meter (766 with a Knick SE 100N electrode, Berlin, Germany). The pH meter was calibrated using buffer solutions with pH values ranging from 4.0 to 7.0.

#### 4.3.2. Viscosity of Ophthalmic Gels

The viscosity of the formulated experimental hydrogels was measured using a vibrating viscometer (Vibro Viscometer SV-10, A&D Company Ltd., Tokyo, Japan). The test material was placed in a special measuring vessel. The vessel was then secured to the instrument’s work surface, and the sensors were immersed in the test gels. The rotational speed of the cylindrical shaft was 10.0 rpm. Each measurement lasted 10 s at three temperature points (4 ± 1 °C, 22 ± 1 °C, 37 ± 1 °C).

#### 4.3.3. Refractive Index of Ophthalmic Gels

The refractive index of the prepared experimental hydrogels was measured at 22 ± 1 °C using a refractometer (“Metler”) according to the manufacturer’s instructions.

### 4.4. Biological Activity Assessment Methods

#### 4.4.1. Determination of Antimicrobial Activity

The evaluation of in vitro antimicrobial activity was performed in accordance with previously published methods [91]. The bacteriological properties of the experimental formulations were evaluated in vitro by the disk diffusion agar method using Mueller–Hinton agar (Biolife Mueller Hinton Agar II, Milan, Italy), Colombian blood agar (CBA, EO Labs, Bonnybridge, Scotland, UK). Mueller–Hinton agar was prepared to Clinical and Laboratory Standards Institute (CLSI) approved standards and dispensed into 10 cm diameter Petri dishes of ~35 mL each. To cultivate *Streptococcus agalactiae*, Colombian blood agar in Petri dishes was purchased from the manufacturer (CBA, EO Labs, Bonnybridge, Scotland, UK). Clinical (*S. aureus*, *B. cereus*, *E. feacalis*, *E. coli*, *Ps. aeruginosa*, *St. agalactiae*) and reference (*S. aureus* (ATCC 25923), *B. cereus* (ATCC 11778), *E. feacalis* (ATCC 29212), *E. coli* (ATCC 25922), *Ps. aeruginosa* (ATCC27853), *St. agalactiae* (ATCC 13813)) strains were used. Suspensions of 0.5 McFarland density were prepared from the clinical isolates and reference strains. All clinical isolates and reference bacterial strains were obtained from the Veterinary Academy of the Lithuanian University of Health Sciences and the Institute of Microbiology and Virology. Clinical bacterial isolates were collected from animals with eye infections. In the study, a single clinical isolate was used for each bacterial species. A total of 50 µL each of clinical and reference strains of *S. aureus*, *B. cereus*, *E. faecalis*, *E. coli*, and *Ps. aeruginosa* were spread on Müller–Hinton agar, and *St. agalactiae* on blood agar. Subsequently, wells (7 mm in diameter) were made in a Petri dish into which 0.1 mL of the test substance was poured. The plates containing the bacteria were kept for 24 h 37 ± 0.5 °C in a thermostat. The antibacterial properties of the experimental medicinal formulations in vitro were evaluated after 24 h of incubation in a thermostat. The diameter of the sterile zone formed around the wells was measured in millimeters. All measurements were performed in triplicate. The studies were conducted using experimental formulations PE1, PE2, PO3, PO4, PE5, PE6, PO7, and PO8, as well as 0.1% and 0.25% diclofenac sodium, 70% ethanol, and organic solvent (PO0) solutions.

#### 4.4.2. Flow Cytometry

The study was conducted using the rabbit corneal epithelial cell line CCL-60 (SIRC) obtained from the American Type Culture Collection (ATCC). The cytotoxicity of the ophthalmic formulations was evaluated by flow cytometry using cells stained with Guava Nexin^®^ reagent. SIRC cells were cultured in Dulbecco’s Modified Eagle Medium (DMEM) supplemented with 10% fetal bovine serum and 1% penicillin–streptomycin at 37 °C in a humidified atmosphere containing 5% CO_2_. For the experiments, cells were seeded in 12-well plates at a density of 5 × 10^4^ cells per well. The cells were exposed to the test substances for 15 min, 2 h, and 4 h. Following treatment, cells were washed twice with phosphate-buffered saline (PBS), detached using trypsin, and collected by centrifugation. Subsequently, the cells were incubated with Guava Nexin^®^ reagent (Millipore, Billerica, MA, USA) for 20 min at room temperature according to the manufacturer’s instructions. Flow cytometric analysis was performed using a Guava^®^ easyCyte™ HT flow cytometer (Millipore, USA), and the data were analyzed with GuavaSoft 2.2.3 InCyte software.

### 4.5. Statistical Analysis

All experiments were performed three times, and the results are presented as the mean ± standard deviation (SD). Statistical analysis was performed using a two-tailed Student’s *t*-test. Differences were considered statistically significant if *p* < 0.05. Statistical analysis and visualization of the data were performed using OriginPro^®^ 2021 (OriginLab, Northampton, MA, USA), IBM SPSS Statistics 27 (SPSS Inc., Chicago, IL, USA), and SigmaPlot 13.0 (Systat Software, San Jose, CA, USA).

## Figures and Tables

**Table 1:** pH and refractive index values.

	PE1	PE2	PO3	PO4	PE5	PE6	PO7	PO8
pH	7.17 ± 0.04	7.06 ± 0.11	6.47 ± 0.22	6.43 ± 0.29	7.19 ± 0.03	7.15 ± 0.11	6.50 ± 0.03	6.43 ± 0.08
Refractive index	1.337	1.338	1.341	1.340	1.337	1.338	1.340	1.341
After 14 days pH	7.14 ± 0.03	7.03 ± 0.10	6.44 ± 0.22	6.40 ± 0.28	7.16 ± 0.03	7.11 ± 0.12	6.48 ± 0.03	6.41 ± 0.08
Refractive index	1.337	1.337	1.340	1.339	1.336	1.339	1.340	1.340

**Table 2:** Antibacterial activity of propolis in ophthalmic formulations.

Bacterial Species	Strain Type	PE Formulations (mm)	Activity	PO Formulations (mm)	Activity	Diclofenac 0.25% (mm)	Activity
*S. aureus*	Clinical	19.66–21.00	++	12.00–17.33	+	18.00	++
*S. aureus*	Reference	20.66–23.33	++	13.66–21.66	++	19.66	++
*S. agalactiae*	Clinical	12.33–18.66	+	11.33–17.00	+	11.00	+
*S. agalactiae*	Reference	12.66–22.33	++	11.66–18.33	+	13.66	+
*B. cereus*	Clinical	15.00–19.66	++	9.66–14.00	+	13.33	+
*B. cereus*	Reference	16.33–20.00	++	11.33–18.33	+	17.33	+
*E. faecalis*	Clinical	–	–	11.33–14.00	+	0	–
*E. faecalis*	Reference	–	–	12.33–15.00	+	0	–
*E. coli*	Clinical	–	–	10.66–12.33	+	0	–
*E. coli*	Reference	–	–	12.00–13.50	+	0	–
*Ps. aeruginosa*	Clinical	–	–	9.33–11.66	+	0	–
*Ps. aeruginosa*	Reference	–	–	10.33–13.00	+	0	–

Summary of antibacterial activity of propolis-based formulations and diclofenac sodium against clinical and reference bacterial strains. Antibacterial activity is expressed as inhibition zone diameter ranges (mm) obtained from three independent measurements (*n* = 3). PE—ethanol-based propolis formulations; PO—choline chloride-based propolis formulations. Activity scale (++ = strong antibacterial activity (≥18 mm), = moderate antibacterial activity (+ = 12–17 mm), — = no antibacterial activity (<12 mm or 0 mm)). *S. aureus* and *P. aeruginosa* were selected for their clinical significance in ophthalmology, while *B. cereus*, *E. faecalis*, *E. coli*, and *St. agalactiae* were included to evaluate efficacy against a broader spectrum of microorganisms associated with more complex eye infections.

**Table 3:** Compositions and physicochemical properties of experimental formulations.

% m/t	PE1	PE2	PO3	PO4	PE5	PE6	PO7	PO8
**PE**	10	10			10	10		
**PO**			10	10			10	10
**DIC Na**	0.1	0.1	0.1	0.1	0.25	0.25	0.25	0.25
**P407**	7		7		7		7	
**Na CMC**		0.25		0.25		0.25		0.25
**Purified water**	up to 100	up to 100	up to 100	up to 100	up to 100	up to 100	up to 100	up to 100

PE—formulations containing ethanolic propolis extract; PO—formulations containing choline chloride-based propolis extract; P407—Poloxamer 407; Na CMC—sodium carboxymethylcellulose; DIC Na—diclofenac sodium.

## Data Availability

The data presented in this study are openly available in the article.
